# Genome engineering *Escherichia coli* for L-DOPA overproduction from glucose

**DOI:** 10.1038/srep30080

**Published:** 2016-07-15

**Authors:** Tao Wei, Bi-Yan Cheng, Jian-Zhong Liu

**Affiliations:** 1 Biotechnology Research Center and Biomedical Center, South China Sea Bio-Resource Exploitation and Utilization Collaborative Innovation Center, School of Life Sciences, Sun Yat-sen University, Guangzhou 510275, China

## Abstract

Genome engineering has become a powerful tool for creating useful strains in research and industry. In this study, we applied singleplex and multiplex genome engineering approaches to construct an *E. coli* strain for the production of L-DOPA from glucose. We first used the singleplex genome engineering approach to create an L-DOPA-producing strain, *E. coli* DOPA-1, by deleting transcriptional regulators (tyrosine repressor *tyrR* and carbon storage regulator A *csrA*), altering glucose transport from the phosphotransferase system (PTS) to ATP-dependent uptake and the phosphorylation system overexpressing galactose permease gene (*galP*) and glucokinase gene (*glk*), knocking out glucose-6-phosphate dehydrogenase gene (*zwf*) and prephenate dehydratase and its leader peptide genes (*pheLA*) and integrating the fusion protein chimera of the downstream pathway of chorismate. Then, multiplex automated genome engineering (MAGE) based on 23 targets was used to further improve L-DOPA production. The resulting strain, *E. coli* DOPA-30N, produced 8.67 g/L of L-DOPA in 60 h in a 5 L fed-batch fermentation. This titer is the highest achieved in metabolically engineered *E. coli* having PHAH activity from glucose.

L-DOPA (3,4-dihydroxyphenyl-L-alanine) is an aromatic compound that is derived from L-tyrosine (Fig. 1). L-DOPA has been used to treat Parkinson’s disease, which is caused by deficiency of the neurotransmitter dopamine. Since Monsanto developed a commercial process for L-DOPA synthesis by asymmetric hydrogenation, L-DOPA has been produced by asymmetric, enzymatic and microbial synthesis^1^. However, the asymmetric synthesis has major disadvantages such as a poor conversion rate and low enantioselectivity. Thus, biotechnology approaches using microorganisms or enzymes have been explored as alternatives. Microorganisms with tyrosinase^2^,^3^,^4^,^5^,^6^,^7^,^8^, tyrosine phenol-lase (Tpl)^9^,^10^,^11^,^12^,^13^ and *p*-hydroxyphenylacetate 3-hydroxylase (PHAH)^14^ activity have been used to produce L-DOPA. However, the microbial fermentations require tyrosine or catechol/pyruvate as substrates, leading to high production costs. Nakagawa *et al*. constructed an *E. coli* expressing *Streptomyces castaneoglobisporus* tyrosinase gene, which can produce 293 mg/L of L-DOPA from glucose^15^. Muñoz *et al*. reported an engineered *E. coli* having PHAH activity, which can produce 1.5 g/L of L-DOPA from glucose^16^. However, the titer of L-DOPA in the engineered *E. coli* is lower than that of the microbial fermentation from tyrosine or catechol/pyruvate. Thus, further work must be carried out to increase L-DOPA production from glucose in *E. coli*.

Genome engineering is a powerful technique to manipulate entire genomes for obtaining desired phenotypes. The singleplex and multiplex genome engineering approaches have been successfully used for strain development^17^,^18^,^19^,^20^,^21^,^22^. Thus, we first focus on increasing the supply of the precursor, tyrosine, by using a singleplex genome engineering approach. We then apply multiplex automated genome engineering (MAGE) to develop an *E. coli* strain overproducing L-DOPA.

## Results and Discussion

**Figure 1:**
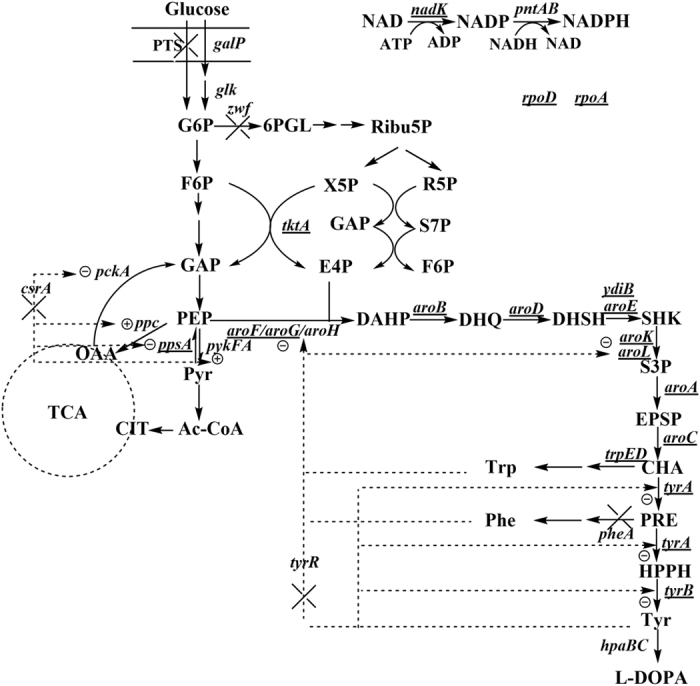
Schematic representation of metabolic pathways involved in L-DOPA biosynthesis and regulation in *E. coli.* The strategies for constructing a genetically defined strain for L-DOPA overproduction are also shown. The ×’s indicate that the genes are deleted. Encircled − or + symbols indicate inhibition or activation, respectively. The genes targeted by MAGE are underlined. PTS: phosphotransferase system; TCA: tricarboxylic acid cycle; G6P: glucose 6-phosphate; 6PBL: 6-phospho D-glucono-1,5-lactone; Ribu5P: D-ribulose 5-phosphate; X5P: D-xylulose 5-phosphate; R5P: D-ribose 5-phosphate; S7P: D-sedoheptulose 7-phosphate; F6P: fructose 6-phosphate; GAP: glyceraldehyde 3-phosphate; E4P: D-erythrose 4-phosphate; PEP: phosphoenolpyruvate; Pyr: pyruvate; Ac-CoA: acetyl-CoA; OAA: oxaloacetate; CIT: citrate; DAHP: 3-Deoxy-arabino-heptulonate 7-phosphate; DHQ: 3-Dehydroquinate; DHSH:3-Dehydroshikimate; SHK: shikimate; S3P: shikimate 3-phosphate; EPSP: 5-enolpyruvyl-shikimate 3-phosphate; CHA: Chorismate; PRE: prephenate; HPPH: 4-hydroxyphenylpyruvate. *galP*: galactose permease gene; *glk:* glucokinase gene; *zwf:* glucose-6-phosphate dehydrogenase gene; *tktA:* transketolase I gene; *pckA*: PEP carboxykinase gene; *ppc:* PEP carboxylase gene; *ppsA:* PEP synthase gene; *pykFA:* pyruvate kinase I/II gene; *aroF, aroG and aroH:* DAHP synthase gene; *aroB:* DHQ synthase gene; *aroD:* DHQ dehydratase; *aroE/ydiB*: shikimate/quinate dehydrogenase gene; *aroA:* 3-phosphoshikimate-1-carboxyvinyltransferase gene; *aroC:* CHA synthase; *tyrA*: CHA mutase/prephenate dehydrogenase gene; *tyrB:* tyrosine aminotransferase gene; *trpED:* anthranilate synthetase gene; *pheA:* prephenate dehydratase gene; *hpaBC*: *E. coli* W *p*-hydroxyphenylacetate 3-hydroxylase gene. *nadK:* NAD kinase gene; *rpoD*: sigma 70 factor gene; *rpoA*: a subunit of RNA polymerase gene; *csrA*: carbon storage regulator A; *tyrR*: tyrosine repressor.

*E. coli* W *hpaBC* has been successfully introduced into *E. coli* to produce L-DOPA from glucose^16^. Figure 1 shows that tyrosine availability should first be increased to improve L-DOPA production from glucose. Successful strategies for engineering *E. coli* strains that can overproduce tyrosine include: (i) improving the carbon flow through the biosynthetic pathway of interest by removing transcriptional and allosteric regulation; (ii) increasing the availability of the direct precursors phosphoenolpyruvate (PEP) and erythrose-4-phosphate (E4P); (iii) preventing loss of carbon to competing pathways; (iv) enhancing the first enzymatic reaction of the shikimate pathway to yield 3-deoxy-D-arabino-heptulosonate-7-phosphate (DAHP); (v) and identifying and relieving rate-limiting enzymatic reactions. Thus, we first used singleplex genome engineering to increase the supply of tyrosine.

### Removal of transcriptional regulators

Tyrosine repressor (TyrR) is a transcriptional dual regulator that represses the transcription of several genes encoding enzymes involved in aromatic acid biosynthesis^23^. Carbon storage regulator A (CsrA) is a regulator of carbohydrate metabolism. CsrA regulates the levels of three enzymes that participate directly in phosphoenolpyruvate (PEP) metabolism. It activates pyruvate kinase (PykF) and represses PEP carboxykinase (PckA) and PEP synthase (PpsA) in *E. coli*^24^. It has been reported that the inactivation of *tyrR* and *csrA* improves aromatic compound production^25^,^26^,^27^,^28^. Thus, we first deleted *tyrR* and *csrA* to obtain *E. coli* AROM-1 (Fig. 1), resulting in a slight increase in L-DOPA production from 138.7 ± 4.9 mg/L to 148.3 ± 11.7 (Table 1). Munoz *et al*. also reported that knocking out *tyrR* enhanced L-DOPA production in *E. coli*^16^.

### Increasing the availabilities of the precursor PEP by altering glucose transport

Increasing PEP availability is a common strategy for engineering *E. coli* strains for the overproduction of aromatic compounds. In *E. coli*, glucose is mainly transported and phosphorylated by the phosphotransferase system (PTS). Under standard growth conditions, 50% of the glycolytic intermediate PEP resulting from the catabolism of glucose is used as the phosphate donor for phosphorylation and translocation by the PTS. The properties of the PTS limit the production of compounds that have PEP as a precursor. Carmona *et al*. suggested that inactivation of the PTS is the primary strategy for engineering *E. coli* to overproduce aromatic metabolites^29^. Thus, we deleted the PTS (*ptsHIcrr*) to further improve L-DOPA production. The inactivation of the PTS increased the L-DOPA titer to 176 ± 3.6 mg/L (Table 1). Non-PEP-mediated glucose transport and phosphorylation systems have successfully been used for the replacement of the PTS to increase PEP availability^30^,^31^,^32^. Thus, we integrated the *galP* and *glk* under the control of the P37 promoter into the *E. coli* knockout strain AROM-2 to obtain *E. coli* AROM-3. The titer of L-DOPA and growth of *E. coli* AROM-3 harboring pQE30-hpaBC showed no significant difference compared to *E. coli* AROM-2 (*p* < 0.05, Table 1).

### Knockout of Glucose-6-phosphate dehydrogenase gene

Glucose-6-phosphate dehydrogenase (encoded by *zwf*) catalyzes the oxidization of glucose-6-phosphate to gluconate-6-phosphate. It has been reported that knocking out *zwf* drives more carbon flux into the Embden-Meyerhof-Parnas (EMP) pathway and tricarboxylic acid (TCA) cycle^33^. They also found that the *zwf* mutant is able to synthesize pentose phosphate (PP) pathway-derived compounds independently from the oxidative part of the PP pathway by directing its carbon flow from the EMP pathway directly into the non-oxidative part of the PP pathway. Thus, we disrupted *zwf* in *E. coli* AROM-3 to obtain *E. coli* AROM-4. *E. coli* AROM-4 (pQE30-hpaBC) produced L-DOPA at 205.3 ± 2.5 mg/L, which was greater than *E. coli* AROM-3 (pQE30-hpaBC) (Table 1). The stoichiometric analysis demonstrated that the yield of the aromatic compound DAHP approaches the theoretical maximum when E4P is provided by the nonoxidative part of the PP pathway and pyruvate is recycled to PEP by PpsA^34^. The improvement of L-DOPA titer after *zwf* deletion was experimentally demonstrated for the first time.

### Removal of competing pathway

Prephenate can be converted into either tyrosine or phenylalanine. To eliminate the loss of prephenate to the competing reaction (phenylalanine biosynthesis), we deleted prephenate dehydratase and its leader peptide genes (*pheLA*) in *E. coli* AROM-4 to obtain *E. coli* TYR-1. The *pheLA* deletion slightly increased the L-DOPA titer to 209.2 ± 0.9 mg/L (Table 1). Some other groups have previously reported that the *pheLA* deletion increases L-tyrosine production^35^,^36^.

### Coordinating expression of the downstream pathway of chorismate

The bifunctional enzyme Chorismate (CHA) mutase/prephenate dehydrogenase, TyrA, catalyzes the first and second step of L-tyrosine biosynthesis (Fig. 1). TyrA catalyzes both reactions in separate domains of the protein, and the CHA mutase/prephenate hydrogenase is feedback-inhibited by L-tyrosine (up to 95% inhibition of the prephenate dehydrogenase and 45% of the CHA mutase activity^28^. Feedback-resistant mutants of the TyrA *E. coli* enzyme have been used for L-tyrosine overproduction^35^,^36^. Thus, TyrA^fbr^ [M53I/A354V] was used to deregulate the feedback inhibition by tyrosine. Substrate channeling is a powerful tool for balancing the expression of genes. It can increase the catalytic efficiency of the sequential reactions in a biosynthetic pathway^37^,^38^. To increase the rate of CHA conversion to L-DOPA, we first fused the *tyrA*^*fbr*^, *tyrB* and *hpaBC* genes with a (G_4_S)_3_ linker, then integrated the fusion protein chimera under the control of the 7P37 promoter into the chromosome of *E. coli* TYR-1 to obtain *E. coli* DOPA-1. *E. coli* DOPA-1 produced 307.4 ± 3.7 mg/L of L-DOPA.

### Multiplex automated genome engineering

MAGE is an efficient and rapid tool for the genome engineering of bacterial strains. We selected *aroF, aroG, aroB, aroD, ydiB, aroE, ppsA, tktA, nadK, aroL, aroK, aroA, tyrA, tyrB* and *tyrA*^*fbr*^ (M53I/A354V) as target sites to tune translation by ribosome binding site (RBS) replacement (Fig. 1). The RBS sequences were designed to be DDRRRRRDDDD (D = A, G, T; R = A, G) with a total pool complexity of 3.5 × 10^5^ (3^6^ × 2^5^ × 15). Six genes (*aroF*^*P148L*^*, aroG*^*D146N*^*, tyrA*^*M53I*^*, tyrA*^*A354V*^*, rpoD*^*D521E*^ and *rpoA*^*V257R*^) were targeted for amino acid mutations in their open reading frames (ORF). The introduction mutations in *aroF, aroG* and *tyrA* were used to remove product feedback inhibition^23^,^26^,^27^,^28^,^35^,^36^. The *rpoD* and *rpoA* mutants have been successfully used to increase tyrosine production^39^. Two genes (*trpD* and *trpE*) were targeted for inactivation by introducing a revertible premature stop codon into each ORF. To increase the MAGE allelic replacement frequency, the methyl-directed mismatch repair protein gene (*mutS*) of *E. coli* DOPA-1 was first deleted to obtain *E. coli* DOPA-2. *E. coli* DOPA-2 (pSIM6) was used as the starting strain for MAGE. After 30 cycles of MAGE, 1.3 × 10^10^ genetic variants (4.3 × 10^8^ bp variations per cycle for 30 MAGE cycles^19^) were generated. According to an allelic replacement efficiency calculation^22^, 30 MAGE cycles generate 2.3% of genomes with at least 3 out of 23 targeted loci and 6.1 × 10^−12^ of genomes with all 23 targeted loci. One hundred clones from the 5th, 10th, 15th, 20th and 25th cycle and 1000 clones from the 30th cycle were screened in deep-well microplate culture. L-DOPA can be easily oxidized to dopachrome and then polymerized nonenzymatically to form the black pigment melanin^40^. Thus, we selected strains that produced darker cultures for further analysis. Darker cultures in the 48-well microplates were selected for HPLC analysis to determine L-DOPA concentration. Six MAGE strains from the 30^th^ cycle showed higher L-DOPA concentrations in the deep-well microplate analysis, and these were further analyzed in shake flasks. Of the six strains, strain 30-30 produced the highest level of L-DOPA, which was 34% higher than that of the starting strain *E. coli* DOPA-2 (Table 2). Table 2 also shows that all MAGE strains produced more tyrosine and total tyrosine plus L-DOPA than the starting strain. The reason may be because the above modification strategies were used to increase the availability of the precursor, tyrosine. Thus, we removed pSIM6 from MAGE strain 30-30 to obtain *E. coli* DOPA-30, which was used as the L-DOPA-producing strain in subsequent tests. After sequencing, we found that three genes have codon mutations in their ORFs (*aroF*: P148L; *tyrA:* M53I and *rpoD:* D521E, Supplementary Table 1). Only 3 modified loci out of 23 targets may be due to the low MAGE allelic replacement frequency (ARF) for multiple targeted loci. Only 2–4 modified targets were also observed in the MAGE lycopene-producer after 35 cycle MAGE based on 20 targets^19^. The ARF may be increased by increasing cycle numbers, Coselection MAGE (CosMAGE)^21^ or CRMAGE^41^. CosMAGE improves the ARF of each target site by around four-fold^21^. CRMAGE increases the efficiency from 6% of traditional MAGE to 66%^41^.

As shown in Table 2, not all of the tyrosine was converted to L-DOPA in *E. coli* DOPA-30. In order to convert all L-tyrosine into L-DOPA, we added a single additional copy of the *hpaBC* into pQE30-hpaBC to obtain pQE30-2hpaBC and transformed the plasmid into *E. coli* DOPA-30. As shown in Table 3, overexpression of *hpaBC* in *E. coli* DOPA-30 indeed increased L-DOPA production, but this strain cannot also convert all the L-tyrosine into L-DOPA. However, the engineered *E. coli* with the *hpaBC* reported by Munoz *et al*. produced few L-tyrosine^16^. Comparing the sequence of the *hpaBC* in pQE30-2hpaBC with that reported by Munoz *et al*.^16^, the 5′-UTR sequence of the *hpaC* has been changed. The change may lead to the imbalanced expression between the *hpaB* and *hpaC*. Is this change resulted in the accumulation of L-tyrosine in the engineered strain? We re-amplified the *hpaBC* operon with the native 5-UTR sequence of the *hpaC* to obtain pQE30-hpaBC_N_. As shown in Table 3, *E. coli* DOPA-30 harboring pQE30-hpaBC_N_ cannot produce L-tyrosine. Thus, the *hpaBC* in *E. coli* DOPA-30 was replaced with the *hpaBC*_*N*_ to obtain *E. coli* DOPA-30N. As shown in Table 3, *E. coli* DOPA-30N cannot also produce L-tyrosine and produced 614.3 mg/L of L-DOPA.

### Fed-batch fermentation

**Figure 2:**
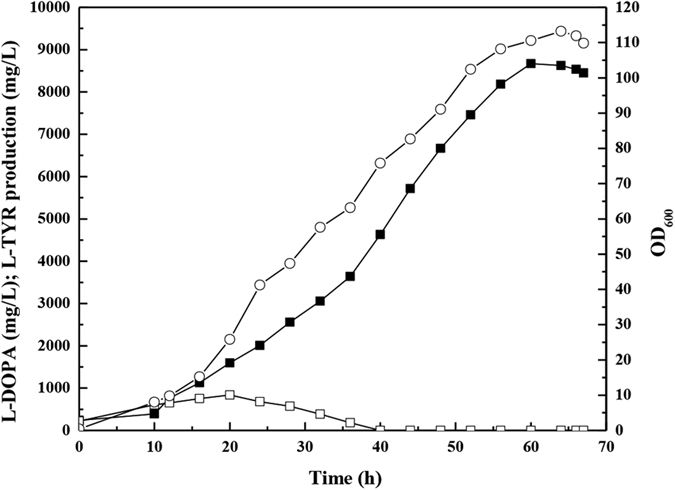
Fed-batch culture of *E. coli* DOPA-30N in a 5 L bioreactor. (○)OD_600_; (◾) L-DOPA concentration; (◽)L-tyrosine concentration. Experiments were conducted in triplicates, and measurements are presented with their means and s.d.

Fed-batch fermentation of *E. coli* DOPA-30N was performed in a 5 L bioreactor. As shown in Fig. 2, the strain produced 8.67 g/L of L-DOPA at 60 h. The OD_600_ of the culture reached 110. The L-DOPA productivity was 144.5 mg/L/h. The L-DOPA yield from glucose was 62.7 mg/g. The titer and yield were 5.7- and 1.2-fold higher than that reported by Muñoz *et al*.^15^, respectively. In addition, it was found that all the L-tyrosine was converted to L-DOPA after 40 h. The similar phenomenon was also observed by Muñoz *et al*.^15^. It indicates that the rate of hydroxylation of L-tyrosine by the HpaBC is slower than the rate of L-tyrosine synthesis. Therefore, the catalytic efficiency of the PHAH encoded by *hpaBC* should be improved.

### Comparison with other microorganisms

L-DOPA production by microorganisms is summarized in Table 4. The L-DOPA titer obtained in this study is higher by a factor of 5.7 than the highest level previously reported using metabolically engineered *E. coli* strain that have PHAH activity from glucose^16^. The value is also higher than that obtained in microorganisms that have tyrosinase activity from tyrosine^2^,^3^,^4^,^5^,^6^,^7^,^8^. However, the value in this study is lower than that obtained in some microorganisms with Tpl activity from catechol and pyruvate^9^,^10^,^12^. It indicates that further works should be carried out for improving L-DOPA production.

Although the L-DOPA titer of our engineered *E. coli* is considerably higher than that previously reported, all of the tyrosine was converted to L-DOPA only after 40 h (Fig. 2). It indicates that PHAH is the rate-limited step for L-DOPA biosynthesis in this strain. The catalytic efficiency of the PHAH encoded by *hpaBC* should be improved. Directed evolution may be used to increase its catalytic efficiency. Because only three targets were found in the MAGE strain (Supplementary Table 1), we can apply other strategies to further enhance the availability of tyrosine, such as upregulating *tktA*, increasing NADPH availability and upregulating *hpaBC*.

In conclusion, we first constructed an L-DOPA-producing *E. coli* strain, DOPA-1, using a singleplex genome engineering approach based on knockouts of genes and integration of the *tyrA*^*fbr*^, *tyrB* and *hpaBC* fusion protein chimera. MAGE based on 23 targets was then used to further improve L-DOPA production, which yielded the strain *E. coli* DOPA-30N. *E. coli* DOPA-30N produced 8.67 g/L of L-DOPA in 60 h in a 5L fed-batch fermentation. This titer is the highest reported in metabolically engineered *E. coli* that has PHAH activity from glucose. This strain, *E. coli* DOPA-30N, can serve as a base strain for developing more efficient strains capable of producing L-DOPA or other aromatic compounds. The rapid and efficient markerless deletion approach using the IPTG-inducible *ccdB* as a counter-selectable marker will be generally useful for gene knockout of *E. coli*.

## Methods

### Strains, plasmids and primers

The strains and plasmids used in this study are listed in Table 5. The primers are listed in Supplementary Table 2.

### Genetic methods

The genes *hpaB* and *hpaC* were amplified from *E. coli* W using the primers hpaB-F/hpaB-R and hpaC-F/hpaC-R, respectively. The *hpaB* fragment was cloned into the SacI/KpnI sites of pQE30 to obtain pQE30-hpaB. The *hpaC* fragment was cloned into the KpnI/SalI sites of pQE30-hpaB to obtain pQE30-hpaBC. The *hpaBC* genes were also amplified from pQE30-hpaBC using the primers hpaBC-F/hpaBC-R and then cloned into the SalI/HindIII sites of pQE30-hpaBC to obtain pQE30-2hpaBC. The *hpaBC* operon was amplied from *E. coli* W using the primers hpaB-F/hpaC-R and then cloned into the SacI/SalI to obtain pQE30-hpaBC_N_.

The knockouts of the *csrA*, *tyrR* and *mutS* genes were carried out according to the one-step inactivity method^42^ with the help of the pSIM6 plasmid^43^ expressing the lambda red recombination system. Gene knockouts were verified by colony PCR using appropriate primers (Supplementary Table 2).

The knockouts of other genes were carried out by a two-step recombination method using lambda red recombination and I-SceI cleavage as described as in Supplementary Fig. 1. The method was first reported by Yu *et al*.^44^. They used *sacB* as the counter-selectable marker. However, the efficiency of the first recombination is very low (24%) because *sacB* generally results in a certain number of false-positive colonies in the screening process due to mutation of *sacB*^45^. Thus, we used the IPTG-inducible *ccdB* gene as the counter-selectable marker. The *ccdB* gene was amplified from pOSIP-CH^46^ using the primers ccdBF/ccdBR, then cloned into the HindIII/XbaI sites of pXMJ19^47^ to obtain pXMJ-ccdB. The plasmid pXMJ-ccdB was digested by HindIII, blunted and self-ligated to obtain pEC-ccdB*. The IPTG-inducible *ccdB* gene was amplified from pXMJ-ccdB* using the primers ccdB*F/ccdB*R, then cloned into pMD18 to obtain pMD-lacI-P_tac_ccdB. A *kan* resistance gene (encoding aminoglycoside 3′-phosphotransferase) containing I-SceI recognition sites was amplified from pK-JL^48^ using the primers kanF/kanR and then cloned into the XhoI/SpeI sites of pMD-lacI-P_trc_ccdB to obtain pMD-ccdBKanS. The *I-Scel* endonuclease gene was synthesized by Suzhou GENEWIZ, Inc. (Suzhou, China) and ligated into pUC57 to obtain pUC57-I-SceI. The *I-Scel* was cut from pUC57-I-SceI by EcoRI/KpnI and cloned into pBAD30^49^ to obtain pBAD30-I-SceI. The arabinose-inducible *I-Scel* was amplified from pBAD30-I-SceI using the primers IsceIF/IsceIR and cloned into the NdeI site of pSIM6^50^ to obtain pSIMIS. The efficiency of the first recombination of the method reached 80.3%, which was much higher than that based on the *sacB* (24%, Supplementary Table 3).

Chromosomal integration was carried out by direct transformation as described by Chen *et al*.^51^ and Huang *et al*.^52^. The *galP* and *glk* genes were amplified from *E. coli* using the corresponding primers and cloned into pZSBP^37^ to obtain pZSBP-galP and pZSBP-glk, respectively. The *glk* gene under the control of the P37 promoter was digested with MluI/SalI from pZSBP-glk, then ligated into MluI/SalI-digested pHKKT5b to yield pHKKT5b-P37-glk. The *galP* gene under the control of the P37 promoter was digested with BglII/SalI from pZSBP-galP, then ligated into BamHI/SalI-digested pHKKT5b-P37-glk to yield pHKKT5b-P37-glk-P37-galP for chromosomal integration of P37-galP-P37-glk. The P37 promoter was amplified from pZSPB using the primers P37F/P37R and assembled into pZSPB by the BglBrick standard approach to produce pZSnP37 (n = 2, 3, 4, 5, 6 or 7), which has a tandem and stronger promoter. The *tyrA* and *tyrB* genes were amplified from *E. coli* using the corresponding primers and cloned into pMD-19T (simple) to obtain pMD-19T-tyrA and pMD-19T-tyrB, respectively. Site-directed mutagenesis was used to remove the BamHI/BglII sites and feedback inhibition of the *tyrA* to obtain pMD-19T-tyrA^fbr^. The *hpaBC* gene was amplified from pQE30-hpaBC using the primers hpaBCF1/hpaBCR2 and cloned into pMD-19T (simple) to obtain pMD-19T-hpaBC. The plasmid pMD-19T-tyrA^fbr^-tyrB-hpaBC containing the tyrA^fbr^-tyrB-hpaBC fusion protein chimera was assembled by the BglBrick standard approach. The fusion chimera fragment was cut from pMD-19T-tyrA^fbr^-tyrB-hpaBC by SphI/ApaI, then ligated into SphI/ApaI-digested pZS7P37 to yield pZS7P37-tyrA^fbr^-tyrB-hpaBC. The *tyrA*^*fbr*^-*tyrB*-*hpaBC* fragment under the control of the 7P37 promoter was cut from pZS.7P37-*tyrA*^*fbr*^-*tyrB*-*hpaBC* by MluI/BamHI, then cloned into the integration expression vector pP21KT5b to yield pP21KT5b-7P37-*tyrA*^*fbr*^-*tyrB*-*hpaBC* for chromosomal integration of 7P37-*tyrA*^*fbr*^-*tyrB*-*hpaBC*.

The replacement of 5′-UTR of the hpaC in *E. coli* DOPA-30 was carried out by the CRIPR-Cas method as described by Jang *et al*.^53^. The sgRNA fragment was amplified from pTargetF using the primers hpaCN20F/hpaCN20R and then cloned into the SpeI/XhoI sites of pTargetF to obtain the sgRNA plasmid pTargetF-hpaC. The target fragment was amplied from pQE30-hpaBC_N_ using the primers hpaB/hpaBC.

### MAGE and Screening of MAGE strains

Oligos were mixed in equimolar amounts to reach a final total oligo concentration of 1 μM. MAGE cycling was performed as previously described^19^,^20^,^21^. In brief, *E. coli* DOPA-3 harboring pSIM6 was grown in a 20-mL conical tube containing 5 mL of LB medium supplemented with 100 μg/mL ampicillin at 30 °C with 200 rpm agitation until the OD_600_ reached 0.5 to 0.7. Then, the cultures were heat-shocked in a shaking water bath at 42 °C for 15 min to induce the expression of λ Red recombination genes (*gam, bet* and *exo*). The cells were then chilled to 4 °C and centrifuged at 11,000 rpm for 30 s at 4 °C. The cultures were washed three times with ice-cold sterile 10% glycerol to remove salts. The cells were resuspended in 50 μL oligo mixture. Electroporation was carried out at 1.8 kV in 1-mm gap cuvettes on a Bio-Rad MicroPulser, BTX ECM-830. Cells were incubated in fresh LB low salt medium at 30 °C until their OD_600_ reached 0.4 to 0.6. The processes were repeated 30 times (30 MAGE cycles). After 5, 10, 15, 20, 25 and 30 cycles, the cells were grown overnight in 50 mL LB low salt medium and stored at −80 °C in a 15% (v/v) glycerol solution.

Cells from the 5th, 10th, 15th, 20th, 25th and 30th cycles were diluted, plated onto LB-agar plates with ampicillin and cultured overnight. Individual colonies were inoculated in individual wells of a 48-well deep-well microplate (4.6 mL) containing 600 mL of the fermentation medium without ascorbic acid and incubated at 30 °C with 200 rpm agitation for 48 h on a Microtron shaker (Infors). Because L-DOPA can be easily oxidized to dopachrome and then polymerized nonenzymatically to form melanin^40^, darker cultures were selected for HPLC analysis to determine L-DOPA concentration. Cultures with higher L-DOPA concentrations in the deep-well microplate analysis were selected for shake flask analysis. In the screening process, the culture temperature was set to 30 °C because the cells harbored pSIM6.

### L-DOPA production in shake flasks

For L-DOPA production, a single colony was inoculated into 5 mL of LB medium in a 20-mL conical tube which was cultured overnight at 37 °C in a rotary shaker at 200 rpm. The overnight seed culture was then inoculated into 50 mL of fermentation medium with a starting OD_600_ of 0.1. The fermentation medium (pH 7.0) contains (g/L): peptone 10, yeast extract 5, NaCl 10, glucose 14, ascorbic acid 0.45 and 10 mL of trace element solution. The trace element solution contains (g/L): FeSO_4_·7H_2_O 10, ZnSO_4_·7H_2_O 2.2, MnSO_4_·4H_2_O 0.58, CuSO_4_·5H_2_O 1, (NH_4_)_6_Mo_7_O_24_·4H_2_O 0.1, Na_2_B_4_O_7_·10H_2_O 0.2 and HCl 10 mL. The main cultures were incubated at 37 °C for 48 h in a rotary shaking incubator at 150 rpm. IPTG was added as an inducer to a final concentration of 0.1 mM after 6 h when needed.

### Fed-batch culture for L-DOPA production

The seed culture produced in 5 mL of LB medium was subcultured in 6 × 50 mL LB medium for 10–12 h with shaking at 200 rpm at 37 °C. The seed culture (~300 mL) was inoculated into a 5 L fermenter (Biostat B5, B. Braun, Germany) containing 3 L of fermentation medium with an initial OD_600_ of approximately 0.4. The fermentation medium (pH 7.0) contains (g/L): peptone 10, yeast extract 5, NaCl 10, glucose 25, (NH_4_)_2_SO_4_ 15, KH_2_PO_4_ 2, MgSO_4_·7 H_2_O 2, CaCl_2_ 14.7 mg, thiamine 0.1 mg, ascorbic acid 1.8, and 1 mL of trace element solution. The trace element solution contains (mg/L): EDTA 8, CoCl_2_·6 H_2_O 2.5, MnCl_2_·4H_2_O 15, CuCl_2_·2H_2_O 1.5, H_3_BO_3_ 3.0, Na_2_MoO_4_·2H_2_O 2.5, Zn(CH_3_COO)_2_·2H_2_O 13.0, Fe(III)citrate 100, thiamine·HCl 4.5. Fermentation was carried out at 37 °C with an airflow of 3 L/min and agitation rate of 600 rpm. IPTG was added as an inducer to a final concentration of 0.1 mM after 24 h. The pH was controlled at 7.0 by automatic addition of NH_4_OH. The feed solution (pH 7.0,) contains (g/L): glucose 500, tryptone 25, yeast extract 50, MgSO_4_·7H_2_O 17.2, (NH_4_)SO_4_ 7.5, ascorbic acid 18. The feed was introduced continuously into the fermenter by using the pH-stat feeding strategy. Once the glucose is exhausted, the pH rises rapidly. When the pH was higher than 7.0 by 0.1 U, the feed was automatically added to the fermenter. A total of 680 mL feed solution was added. Samples were periodically withdrawn, and the following parameters were measured: OD_600_, residual glucose concentration, tyrosine concentration and L-DOPA concentration. Fermentation experiments were carried out in triplicate.

### Analytical methods

Growth was monitored by measuring the optical density at 600 nm. Tyrosine and L-DOPA in the supernatants were analyzed using a Shimadzu HPLC system (LC-20 A, Shimadzu, Japan) equipped with an Inertsil ODS-SP column (5 μm, 4.6 × 150 mm, GL Sciences Inc., Tokyo, Japan). The mobile phase was 0.2% TFA in 40% methanol, with a flow rate of 0.5 mL/min, at 30 °C. A photodiode array detector (SPD-M20A) operating at 280 nm was used, and a standard curve was constructed from serial dilutions of a standard stock solution. Glucose concentration was determined by using glucose oxidase and a glucose assay kit (Shanghai Rongsheng Biotech Corporation, Shanghai, China).

## Additional Information

**How to cite this article**: Wei, T. *et al*. Genome engineering *Escherichia coli* for L-DOPA overproduction from glucose. *Sci. Rep.* **6**, 30080; doi: 10.1038/srep30080 (2016).

## Supplementary Material

Supplementary Information

## Figures and Tables

**Table 1:** L-DOPA production in different *E. coli* strains*.

Strain	Genetic modification of the host strain	OD_600_	Tyrosine (mg/L)	L-DOPA (mg/L)
*E. coli* BW25113 (pQE-hpaBC)		5.55 ± 0.08	292.5 ± 5.2	138.7 ± 4.9
*E. coli* AROM-1 (pQE-hpaBC)	*E. coli* BW25113, ∆*tyrR*, ∆*csrA*	6.13 ± 0.06	263.5 ± 60.8	148.3 ± 11.7
*E. coli* AROM-2 (pQE-hpaBC)	*E. coli* BW25113, ∆*tyrR*, ∆*csrA*, ∆*ptsHI*, ∆*crr*	4.57 ± 0.04	366.2 ± 11.8	176.0 ± 3.6
*E. coli* AROM-3 (pQE-hpaBC)	*E. coli* BW25113, ∆*tyrR*, ∆*csrA*, ∆*ptsHI*, ∆*crr*, P_37_-*galP*-P_37_-*glk*	4.56 ± 0.07	304.0 ± 25.5	173.9 ± 11.7
*E. coli* AROM-4 (pQE-hpaBC)	*E. coli* BW25113, ∆*tyrR*, ∆*csrA*, ∆*ptsHI*, ∆*crr*, P_37_-*galP*-P_37_-*glk*, ∆*zwf*	4.50 ± 0.03	256.9 ± 7.8	205.3 ± 2.5
*E. coli* TYR-1 (pQE-hpaBC)	*E. coli* BW25113, ∆*tyrR*, ∆*csrA*, ∆*ptsHI*, ∆*crr*, P_37_-*galP*-P_37_-*glk*, ∆*zwf*, ∆*pheLA*	4.59 ± 0.05	256.6 ± 4.8	209.2 ± 0.9
*E. coli* DOPA-1	*E. coli* TYR-1, attP_P21_::7P37-*tyrA*^*fbr*^*-tyrB-hpaBC* fusion protein chimera	4.24 ± 0.09	241.3 ± 6.2	307.4 ± 3.7

^*^Experiments were conducted in triplicates, and measurements are presented with their means and s.d.

**Table 2:** L-DOPA production in MAGE strain harboring pSIM6*.

Strain	Tyrosine (mg/L)	L-DOPA (mg/L)	Total L-DOPA plus tyrosine (mg/L)
*E. coli* DOPA-2	236.3 ± 6.2	287.7 ± 3.7	524.0
30–6	384.4 ± 1.9	269.3 ± 5.1	653.7
30–12	355.6 ± 8.0	289.9 ± 6.8	645.5
30–27	373.9 ± 5.9	267.1 ± 3.8	641.0
30–30	372.4 ± 35.9	386.2 ± 40.0	758.6
30–52	382.4 ± 10.6	297.9 ± 4.0	680.3
30–71	376.6 ± 8.3	279.3 ± 6.7	655.9

^*^Experiments were conducted in triplicates, and measurements are presented with their means and s.d.

**Table 3:** Effect of overexpression of *hpaBC* on L-DOPA production*.

Strain	OD_600_	Tyrosine (mg/L)	L-DOPA (mg/L)
*E. coli* DOPA-30 (pQE30)	4.87 ± 0.06	462.7 ± 7.0	210.2 ± 6.9
*E. coli* DOPA-30(pQE30-2hpaBC)	7.10 ± 0.15	546.8 ± 10.4	490.3 ± 8.3
*E. coli* DOPA-30 (pQE30-phaBC_N_)	5.08 ± 0.02	0.0 ± 0.0	650.3 ± 23.6
*E. coli* DOPA-30N	4.87 ± 0.06	0.0 ± 0.0	614.3 ± 19.1

^*^Experiments were conducted in triplicates, and measurements are presented with their means and s.d.

**Table 4:** L-DOPA production in different microorganisms.

Microorganism	Substrate	Enzyme	L-DOPA (g/L)	Reference
*Acremonium rutilum*	tyrosine	Tyrosinase	0.89	2
*Aspergillus oryzae*	tyrosine	Tyrosinase	1.69	3,4
*Yarrowia lipolytica* NRRL-I43	tyrosine	Tyrosinase	2.96	5
*Bacillus sp.* JPJ	tyrosine	Tyrosinase	0.5	6
*Brevundimonas sp.* SGJ	tyrosine	Tyrosinase	3.81	7,8
*E. coli*	glucose	Tyrosinase	0.293	15
*Erwinia herbicola*	catechole, pyruvate	Tpl	11.1 g/L/h	9
*E. coli*	catechole, pyruvate	Tpl	20.7	10
*E. coli*	catechole, pyruvate	Tpl	29.8	12
*Pseudomonas aeruginosa*	catechole, pyruvate	Tpl	2.76	13
*E. coli*	tyrosine	PHAH	9.47	14
*E. coli*	glucose	PHAH	1.51	16
*E. coli* DOPA-30N	glucose	PHAH	8.67	This study

**Table 5:** Strains and plasmid used in this study.

Strains/Plasmids		Reference
Strain		
*E. coli* BW25113	*lacI*^q^*rrnB*_T14_*ΔlacZ*_WJ16_*hsdR514 ΔaraBAD*_AH33_*ΔrhaBAD*_LD78_	42
*E. coli* AROM-1	*E. coli* BW25113, ∆*tyrR*, ∆*csrA*	This study
*E. coli* AROM-2	*E. coli* AROM-1, ∆*ptsHI*, ∆*crr*	This study
*E. coli* AROM-3	*E. coli* AROM-2, P_37_-*galP*-P_37_-*glk*	This study
*E. coli* AROM-4	*E. coli* AROM-3, ∆*zwf*	This study
*E. coli* TYR	*E. coli* AROM-4, ∆*pheLA*	This study
*E. coli* DOPA-1	L-DOPA producer, *E. coli* TYR derivative integrated the *tyrA*^*fbr*^*, tyrB* and *hpaBC* fusion protein chimera under the control of 7P37 promoter	This study
*E. coli* DOPA-2	*E. coli* DOPA-1, ∆*mutS*	This study
*E. coli* DOPA-30	MAGE strain with the artificial 5′-UTR sequence of the *hpaC*	This study
*E. coli* DOPA-30N	MAGE strain with the native 5′-UTR sequence of the *hpaC*	This study
Plasmid		
pQE30	Expression vector, T5 promoter, pBR322 *ori*, Amp^r^	Invitrogen
pQE30-hpaBC	pQE30 containing *E. coli* W *hpaBC*	This study
pQE30-2hpaBC	pQE30 containing 2 copies of *E. coli* W *hpaBC*	This study
pOSIP-CH	Integration vector, HK022 integrase, attP_HK022_ aite, *ccdB* gene, cat^r^	46
pXMJ19	*C*. *glutamicum*-*E. coli* shuttle expression vector, P_tac_, IPTG inducible, cat^r^; GenBank No. AJ133195	47
pK-JL	pK18*mobsacB* derivative, *sacB* under the control of the *tac-M* promoter, Kan^r^	48
pMD-ccdBKanS	*ccdB-kan-IsceI* cassette	This study
pBAD30	Expression vector, P_BAD_ promoter, arabinose induction, pACYC184 *ori*, Amp^r^	49
pBAD30-I-SceI	pBAD30 derivate with the I-SceI endonuclease gene	This study
pSIM6	pSC101 replicon^ts^ P_L_-*gam-bet-exo cI*857, Amp^r^	50
pSIMIS	pSIM6 derivative with the arabinose-inducible I-SceI endonuclease gene	This study
pZSBP	Biobrick vector, P37 promoter, pSC101 *ori*, Kan^r^	38
pZSBP-P37-glk	pZSBP derivative with the *glk* under the control of the P37 promoter, respectively	This study
pZSnP37	pZSBP derivative with the nP37 promoter (n = 2,3,4,5,6, or 7)	This study
pZSBP-P37-galP	pZSBP derivative with the *galP* under the control of the P37 promoter	This study
pHKKT5b	Integration expression plasmid, attPHK site, P_T5_ promoter, Kan^r^	52
pHKKT5b-P37-glk	pHKKT5b derivative with the *glk* under the control of the P37 promoter	This study
pHKKT5b-P37-galP-P37-glk	pHKKT5b derivative with the *galP* and *glk* under the control of the P37 promoter, respectively	This study
pP21KT5b	Integration expression plasmid, attPP21 site, P_T5_ promoter, Kan^r^	52
pP21KT5b-7P37-tyrA^fbr^-tyrB-hpaBC	pP21KT5b derivative with the *tyrA*^*fbr*^-*tyrB*-*hpaBC* fusion chimera under the control of the 7P37 promoter	This study
pCas	repA101(Ts) *ori*, kan^r^, Pcas-*cas9*, ParaB-Red, lacIq, Ptrc-sgRNA-pMB1	53
pTargetF	sgRNA plasmid, pMB1 *ori*, Spe^r^	53
pTagetF-hpaC	sgRNA-hpaC plasmid, pMB1 *ori*, Spe^r^	
