# Supplementary material for: Genome engineering Escherichia coli for L-DOPA overproduction from glucose
Source: Sci Rep. 2016 Jul 15;6:30080. doi: 10.1038/srep30080 (PMC4945936; doi:10.1038/srep30080)
Supplement: Supplementary Information [file srep30080-s1.doc]

**Genome engineering *Escherichia coli* for L-DOPA overproduction from glucose**

*Tao Wei, Bi-Yan Cheng**, Jian-Zhong Liu**

*Biotechnology Research Center and Biomedical Center,* *South China Sea Bio-Resource Exploitation and Utilization Collaborative Innovation Center, School of Life Sciences, Sun Yat-sen University, Guangzhou 510275, China*

# Supplementary Table 1 Genomic mutations found in MAGE strain *E. coli* DOPA-30

| No. | Gene name | Type of change | Genomic locus^*^ | mutation |
| --- | --- | --- | --- | --- |
| 1 | *aroF* | ORF | 2738741..2738739 | CCG->CTG, P148L |
| 2 | *tyrA* | ORF | 2737933..2737931 | ATG->ATT, M53I |
| 3 | *rpoD* | ORF | 3212629..3212631 | GAT->GAG, D521E |

^*^Genomic loci reference the *E. coli* MG1655 genome position

# Supplementary Table 2 Primers used in this study

| Name | Sequence | Purpose |
| --- | --- | --- |
| Gene amplification | |  |
| hpaB-F | CGAGCTCTTTCGGAATTAAGGAGGTAATAAATATGAAACCAGAAGATTTCCGCGCCAGCACCCAAC (SacI) | For the *hpaB* |
| hpaB-R | ACAGGTACCTTATTTCAGCAGCTTATCCAG (KpnI) |  |
| hpaC_F | TCAGGTACCTTTCGGAATTAAGGAGGTAATAAATATGCAATTAGATGAACAACGC (KpnI) | For the *hpaC* |
| hpaC-R | GACGTCGACTTAAATCGCAGCTTCCATTTC (SalI) |  |
| hpaBC-F | TTTGTCGACTTTCGGAATTAAGGAGGTAAT (SalI) | For the *hpaBC* |
| hpaBC-R | TTTAAGCTTTTAAATCGCAGCTTCCATTTCC (HindIII) |  |
| P37F | CTGAATTCTCCAGATCTCTTACATGAAAAAGGT | For P37 promoter |
| P37R | CAGGATCCGAAAAATGACATATAC |  |
| tyrAF | CTGAATTCTCCAGATCTATATAGCATGCTTTCGGAATTAAGGAGGTAATAAAT (EcoRI, BglII) | For the *tyrA* |
| tyrAR | CAGGATCCTCCTCCCCCTGAGCCGCCACCTCCCTGGCGATTGTCATTCGCC (BamHI) |  |
| M1F new | AGGCATCTATTTTGGCCTCGCGTCGTGCAG | For the *tyrA*(M53I) |
| M1R new | CGCGCTCCGGAACATAAATAGGCAGT |  |
| M2F new | TCGGCGATTACGTACAGCGTTTTCAGAGTG | For the *tyrA*(A354V) |
| M2R new | ACCAGTGCTCCACCTTGCGGAAACTGTC |  |
| M4F | GGTGTACCGCCGGATCTGATTGAG | Romoval of BglⅡin the *tyrA* |
| M4R | CTCAATCAGATCCGGCGGTACACC |  |
| M5F | CTGTTTGCTCAGGACCCGCAGCTT | Romoval of BamHⅠin the *tyrA* |
| M5R | AAGCTGCGGGTCCTGAGCAAACAG |  |
| tyrBF | CTGAATTCTCCAGATCTGGGGGGGGAGGAAGCGTGTTTCAAAAAGTTGACGCCTAC (EcoRI, BglII) | For the *tyrB* |
| tyrBR | CAGGATCCACCACCGCCGCTTCCACCTCCACCCATCACCGCAGCAAACGCCTTTG (BamHI) |  |
| hpaBCF1 | CTGAATTCTCCAGATCTGGCGGCGGTGGTTCGATGAAACCAGAAGATTTCCGC (EcoRI, BglII) | For the *hpaBC* |
| hpaBCR2 | ACGGATCCTTTGGGCCCTTAAATCGCAGCTTCCATTTC (BamHI) |  |
| ccdBF | CCCAAGCTTTTTCGGAATTAAGGAGGTAATAAATATGCAGTTTAAGGTTTACAC (HindIII) | For *ccdB* |
| ccdBR | TGCTCTAGATTATATTCCCCAGAACATCAGG (XbaI) |  |
| ccdB*F | GCTCATAGACCGTATCCAAAGCATC |  |
| ccdB*R | CGGACTAGTCCGCTCGAGTTATATTCCCCAGAACATCAGG (SpeI, XhoI) | For IPTG-inducible *ccdB* |
| kanF | CCGCTCGAGCCGGAATTGCCAGCTGG (XhoI) | For *kan* |
| kanR | GGACTAGTATTACCCTGTTATCCCTAAGTCCCGCTCAGAAGAA (SpeI) |  |
| IsceIF | GGAATTCCATATGTCCGTCAAGCCGTCAATTGTCTG (NdeI) | For I-SceI |
| IsceIR | GGAATTCCATATGTTATTATTTCAGAAAGGTCTCGC (NdeI) |  |
| glk-F | GCTAGCTTTCGGAATTAAGGAGGTAATAAATGCATGCTAATGACAAAGTATGCATTAG TCGG (NheI, SphI) | For the *glk* |
| glk-R | GGTACCTTACAGAATGTGACCTAAGGTCT (KpnI) |  |
| galP-F | GCATGCTAATGCCTGACGCTAAAAAAC (SphI) | For the *galP* |
| galP-RV | GGGCCCTTAATCGTGAGCGCCTATT (ApaI) |  |
| Gene knockout | |  |
| csrAF | atgctgattctgactcgtcgagttggtgagaccctcatgattggggatgaAGCGATTGTGTAGGCTGGAG | For the *csrA* deletion |
| csrAR | TTAGTAACTGGACTGCTGGGATTTTTCAGCCTGGATACGCTGGTAGATCTACGGCTGACATGGGAATTAG |  |
| tyrRF | atgcgtctggaagtcttttgtgaagaccgactcggtctgacccgcgaattAGCGATTGTGTAGGCTGGAG | For the *tyrR* deletion |
| tyrRR | TTACTCTTCGTTCTTCTTCTGACTCAGACCATATTCCCGCAACTTATTGGACGGCTGACATGGGAATTAG |  |
| cysK-F | CGACCTAGGCTGTTGCCGCGGCGTT (AvrII) | For the PTS deletion |
| cysK-R | TGAAGATCTAGGCCACAAAAAAGCAC (BglII) |  |
| pdxK-F | CTAGGGCCCTTATGCTTCCGCCAGC (ApaI) |  |
| pdxK-R | TCCCCCGGGCTGACCTATTGATCATG (SmaI) |  |
| SKScrr-F | GGATCCTGAGCTGTTGACAATTAAT (BamHI) |  |
| SKScrr-R | TCTAGAAAAAATGGCGCCGATGGGCGCCATTTTTCACTGCGGCAAGAATTACTTCTATTACCCTGTTATCCCTA (XbaI) |  |
| zwf-UPF | CGGAATTCCGCAGAGGTGAGGGCAATCACCATG (EcoRI) | For the *zwf* deletion |
| zwf-UPR | GAAGATCTGTCATTCTCCTTAAGTTAACTAACC (BglII) |  |
| zwf-DNF1 | CGGGATCCAAGAAAATTACAAGTATACCCTGGCTTAAGTACCGGGTTAGTTAACTTAAG (BamHI) |  |
| zwf-DNF2 | TACCGGGTTAGTTAACTTAAGGAGAATGACTATCTGCGCTTATCCTTTATGGTTAT |  |
| zwf-DNR | TGCTCTAGAGAAGAGCCTGGCAACTGCATCCC (XhoI) |  |
| pheLA-UPF | CGAGCTCCGAAAATCATCGCCGCAAACAGCAG (SacI) | For the *pheLA* deletion |
| pheLA-UPR | TGCTCTAGAAAAAAACGCGCCCGAAGGCG ((XhoI) |  |
| pheLA-DNF1 | ACGCGTCGACTGAAGAAGAGTAGTCCTTTATATTGAGTGTATCGCCAACGCGCCTTCG (SalI) |  |
| pheLA-DNF2 | ATCGCCAACGCGCCTTCGGGCGCGTTTTTTTGAAAAGGTGCCGGATGATGTG |  |
| pheLA-DNR | AAACTGCAGGGAATGGTGATTGTTAGTGTGCC (PstI) |  |
| mutSF | ATGAGTGCAATAGAAAATTTCGACGCCCATACGCCCATGATGCAGCAGTAGCGATTGTGTAGGCTGGAG | For the *mutS* deletion |
| mutSR | TTACACCAGGCTCTTCAAGCGATAAATCCACTCCAGCGCCTGACGCGGGACGGCTGACATGGGAATTAG |  |
| hpaCN20F | AATACTAGT GATGGCCAGCCTGTCGGCAGGTTTTAGAGCTAGAAATAGC (SpeI) | For replacing the 5-UTR of the *hpaC* |
| hpaCN20R | GATGGCCAGCCTGTCGGCAG (XhoI) |
| Clone PCR for diagnosis | |  |
| csrF | ATCAACAGAAGAATGGAGGT |  |
| csrR | AATATAAGCGTCAGGCAATG |  |
| tyrF | CCGTCTTTGTGTCAATGATT |  |
| tyrR | GTGCCGTTGTGGTTATTG |  |
| BLP1 | GTTATCGCCTGTATGAACCTG |  |
| BLP4 | GTAGAACTACCACCTGACCG |  |
| AHP2 | ACACTTAACGGCTGACATGG |  |
| AHP3 | AACGAGTATCGAGATGGCAC |  |
| mutSUPF | AAACTGCAGCGGATGACGCCACAGCATTCTC |  |
| mutSDNR | AAGAATTCATTCACTCCCCGGATAATCTGC |  |
| hpaB | TCGGTGAAGTGGTAGCGTGG |  |
| hpaBC | ACGGATCCTTTGGGCCCTTAAATCGCAGCTTCCATTTC |  |
| For MAGE | |  |
| aroF-RBS (b2601) | C*G*GTAATATGTACGTTATTCAGCGCGTCTTTTTGCATGATHHHHYYYYYHHTTATG CTCGTTTGCG ATAGTTGATC CTCAGCGAGG ATGA | |
| aroF (P148L) | T*T*GCTGACCAGCTAAACAGATCGCCCAGGTATTGCGGGCTATTCAGATCTAACGCTTCCGTCGCCAGTGGCAGTCCCATATTCACCAGCT | |
| aroG-RBS (b0754) | T*T*TCTTTGATGCGTAAATCGTCGTTCTGATAATTCATGTCHHHHYYYYYHHTGC CATACTTATC TTAGTGAATG CAATACTTCC AATCTA | |
| aroG (Asp146Asn) | C*G*CCCCAGCTCATCAGGTCAGCGAGATATTGTGGGGTGATCATATTGAGAAACTCACCTGCCGCTGGCAGACCGCTGTCGTTAATATCAA | |
| aroB-RBS (b3389) | A*A*CTACGTTCCCCGAGAGTAACGACAATCCTCTCCATAACHHHHYYYYYHHCTTAATTACTGTACCCGCAGACGAGTGTATATAAAGC CA | |
| aroD-RBS (b1693) | A*G*GTCATGGGGTTCGGTGCCTGACAGGCTGACCGCGTGCDDRRRRRDDDDAAAATGAAAACCGTAACTGTAAAAGATCTCGTCATTGGTA | |
| ydiB-RBS (b1692) | A*A*ATTTTGCACCCGGTTAAACACAATTAAGCATAGAGGTDDRRRRRDDDDATTATGGATG TTACCGCAAA ATACGAATTG ATTGGGTTGA | |
| aroE-RBS (b3281) | G*G*GCTATCGGATTACCAAAAACAGCATAGGTTTCCATTATHHHHYYYYYHHCGAAACA GTTCACCCGT CAGGGCATCG CGGATTTCTG AA | |
| ppsA-RBS (b1702) | A*C*CAAAGCACCAGCGGTGACGAGCCATTGTTGGACATCGAHHHHYYYYYHHGTGATAA ATGAACGGTT TGAGAAACACATTTCTGCGC AT | |
| tktA-RBS (b2935) | C*A*CGAATAGCATTGGCAAGCTCTTTACGTGAGGACATTTTHHHHYYYYYHHGGATGATGAAGGGCACGCCCTTAACGACTTGACGACAGC | |
| nadK-RBS (b2615) | C*A*AGGGAAGCACTCACATTGTCATCAATCTTCGCAACAADDRRRRRDDDDAAAATGAATAATCATTTCAAGTGTATTGGCATTGTGGGAC | |
| aroL-RBS (b0388) | A*G*CCCCGAGGCCCGATCAGAAAAAGAGGTTGTGTCATCGTHHHHYYYYYHHAATAGGTCGCAATGCGGCGAAAGCCGGTGTCATGAGAAT | |
| aroK-RBS (b3390) | T*A*GGCCCAACCAGAAAGATATTGCGTTTCTCTGCCATTTTHHHHYYYYYHHTAAGACTATTCGTTAATGATAAACCCGCTTCGCTCAGAG | |
| aroA-RBS (b0908) | C*G*ACACGAGCGATGGGTTGTAACGTCAGGGATTCCATGAAHHHHYYYYYHHACAACAGAAATA AAAACCCCAC AGGCTGGCTG TGGGGAT | |
| tyrA-RBS (b2600) | C*A*ATTTGATCGCGTAATGCGGTCAATTCAGCAACCATAATHHHHYYYYYHHTTAAGCCA CGCGAGCCGT CAGCTGCCCG TTCAGATCCT G | |
| tyrA(M53I) | G*T*ACACCCAGAGCTTCCGCCTCTGCACGACGCGAGGCCAAaATAGATGCCTCGCGCTCCGGAACATAAATAGGCAGTCCAAAGCGGCTTT | |
| tyrA(A354V) | T*C*ATTCGCCTGACGCAATAACACGCGGCTTTCACTCTGAAAACGCTGTaCGTAATCGCCGAACCAGTGCTCCACCTTGCGGAAACTGTCA | |
| tyrB-RBS (b4054) | T*C*GGGTCGCCAGCGTAGGCGTCAACTTTTTGAAACATGCGHHHHYYYYYHHGGTTTACGGGCAGGTGGTTAAAACACAATAAACCGGAAG | |
| rpoD(D521E) (b3067) | G*G*AAACGCCGATCGGTGATGATGAAGATTCGCATCTGGGGGAGTTCATCGAGGATACCAC CCTCGAGCTG CCGCTGGATT CTGCGACCAC | |
| rpoA(V257R) (b3259) | T*T*TAAGGCAGTTAGCAGAGCGGACAGTCAATTCCAGATCGTCACGAGGGCGCAGCAGGATCGGATCGAACTCTGGTTTCTCTTCTTTCAC | |
| trpD-stop (b1263) | G*G*GCATAACGTGGTGATTTACCGCAACCATATTCCGGCGTAAACCTTAATTTAACGCCTGGCGACCATGAGCAATCCGGTGCTGATGCTT | |
| trpE-stop (b1264) | T*A*GAGAATAACAATGCAAACACAAAAACCGACTCTCTAACTGCTAACCTGCTAAGGCGCTTATCGCGACAATCCCACCGCGCTTTTTCAC | |
| tyrA’-RBS | C*A*ATTTGATCGCGTAATGCGGTCAATTCAGCAACCATATTHHHHYYYYYHHTATTACCTCCTTAATTCCGAAAGCATGCTTTTGCTAGCT | |
| Sequencing primers for MAGE | | |
| | aroFMAs | ACGCGAGGCCAACATAGATG | | --- | --- | | ACGCGAGGCCAACATAGATG | |
| | aroFMAa | CAGTAAAGCCAACAAGGG | | --- | --- | | CAGTAAAGCCAACAAGGG | |
| aroGMAs | CATAGGATGCTCCTGTTATG | |
| aroGanti | CTAGGTACCTTACCCGCGACGCGCTTTTACT | |
| aroBMAs | TTCCGCTCGTGGCGTTGTCG | |
| aroBMAa | TTACGCTGATTGACAATCGG | |
| aroDMAs | CGATGGTGCTGTTAGGGGCC | |
| aroDMAa | GGCACTCGTCCATTAATACG | |
| ydiBMAs | CGTTTCGGTCATGTCAGAGT | |
| ydiBMAa | CGCTGCGCGAAGGCGAGGGC | |
| aroEMAs | GCCAGGTCCTGTCACCTTTG | |
| aroEanti | CAGGATCCGCCCCCACCACTCCCTCCGCCCCCCGCGGACAATTCCTCCTGCAAT | |
| ppsAMAs | AGCGTAGAACGTTATGTCTG | |
| ppsanti | CTGAGCTCTTATTTCTTCAGTTCAGCCAG | |
| tktAMAs | ACCGTTGCCACGCTCATTGC | |
| tktAanti | TGACTGCAGTTACAGCAGTTCTTTTGCTTTC | |
| nadKMAs | GCTTCAGCCAGCTGAGCTTC | |
| nadKanti | CGAGCTCTTAGAATAATTTTTTTGACCAGCCG | |
| aroLMAs | GCGCCTGACGATGCGCGATT | |
| aroLanti | CAGGATCCCCCGCCTCCCGAACCCCCCCCGCCACAATTGATCGTCTGTGCCAG | |
| aroKMAs | TCCAGTGAGTAAACAGCCGT | |
| aroKanti | CTAGTCGACTTAGTTGCTTTCCAGCATGTG | |
| aroAMAs | GCAATGACGTGGCGAAAGCT | |
| aroAanti | CTGGATCCAAACTGCAGTCAGGCTGCCTGGCTAATC | |
| tyrAMAs | CATGTGATCCTGCGCGGTGG | |
| tyrAanti | CAGGATCCTCCTCCCCCTGAGCCGCCACCTCCCTGGCGATTGTCATTCGCC | |
| tyrBMAs | GAACGTATCGCTGAAATGAC | |
| tyrBanti | CAGGATCCACCACCGCCGCTTCCACCTCCACCCATCACCGCAGCAAACGCCTTTG | |
| rpoDMAs | GTTGAAGCGAACTTACGTCT | |
| rpoDMAa | CGATTAATCGTCCAGGAAGC | |
| rpoAMAs | AACCTGCCATATTGCGGAAC | |
| rpoAMAa | CCGATGAGAACGCGTCTATT | |
| trpDMAs | CAGAATCGGTTGCAGCGTGT | |
| trpEMAa | GCGAGGAACTCACACATTAG | |
| BLP1 | GTTATCGCCTGTATGAACCTG | |
| AHP2 | ACACTTAACGGCTGACATGG | |

Restriction enzyme sites are underline; * indicates phosphorothiolated bond

# Supplementary Fig. 1


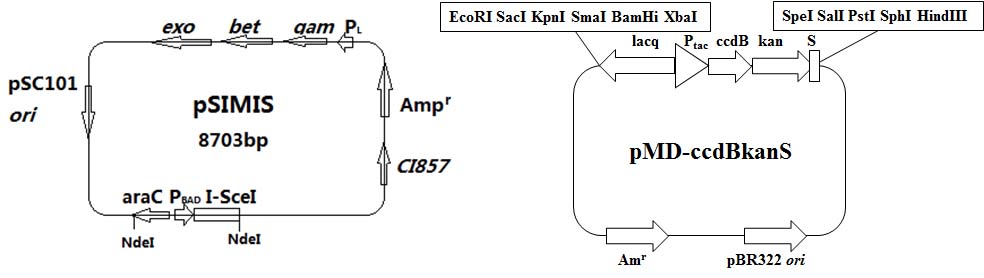


(A)


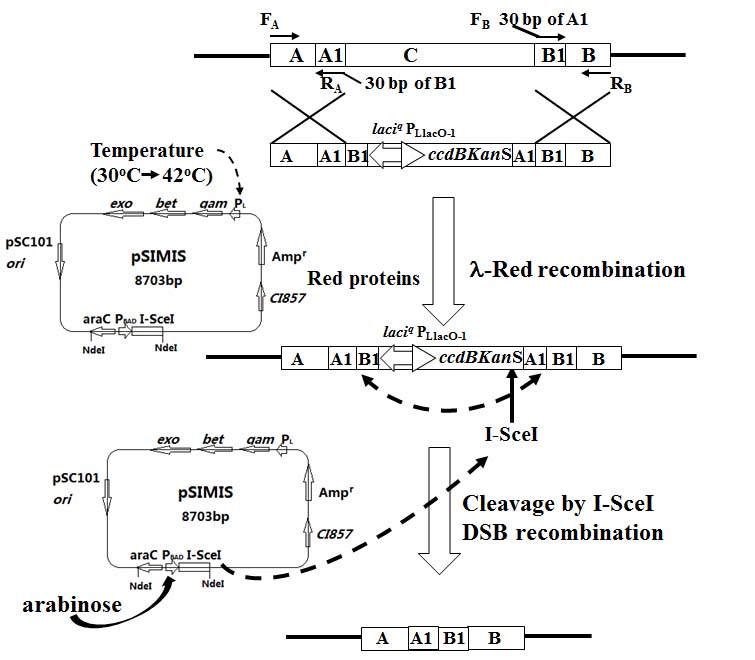


(B)

Supplementary Fig. 1 Description of markerless deletion based on lambda red recombination and I-SceI cleavage. (A) Plasmids used in markerless deletion. (B) Schematic representation of markerless deletion system by using lambda red recombination and I-SceI cleavage. (i) To delete the *E. coli* chromosomal targeted region C, the left and right homologous arms were separately amplified by PCR using primers F_A_/R_A_ or F_B_/R_B_, and then cloned into the left or right MCS of pMD-ccdBkanS. (ii) A linear DNA cassette containing the left arm, ccdBkanS and right arm was obtained by restriction enzymatically digesting or PCR. (iii) The linear DNA cassette was electroported into pSIMIS-containing *E. coli* cells. (iv) The cassette could replace a target genomic segment with help of the λ-Red proteins after temperature induction. Deletion mutants were selected as Kan^r^ and IPTG^S^. (v) The I-SceI endonuclease in pSIMIS was induced by 10 mM arabinose to remove the introduced selection marker. As a result, the chromosome was cleaved at the I-SceI recognition site (S). Then, the double-stranded break (DSB)-mediated intramolecular recombination between the two homology arm (A1B1) led to the removal of the selection marker, yielding a clean, markerless deletion strain.

# Supplementary Table 3 Efficiency of recombination

| Target region | Length (kb) | Right end | Left end | Cassette | First recombination | | | Sencond recombination | | |
| --- | --- | --- | --- | --- | --- | --- | --- | --- | --- | --- |
|  |  |  |  |  | Kan^r^ | IPTG^S^/Kan^R^  (%) | PCR check | IPTG^R^ | Kan^S^/IPTG^R^  (%) | PCR check |
| *zwf* | 1.47 | 1934839 | 1936314 | ccdBKanR | 199 | 85.47 | 11/11 | 50 | 100 | 11/11 |
| *pheLA* | 1.36 | 2737543 | 2738905 | ccdBKanR | 8 | 62.50 | 11/11 | 33 | 100 | 11/11 |
| B4626-b0354 | 114.16 | 262374 | 376535 | ccdBKanR | 28 | 92.86 | 11/11 | 33 | 100 | 11/11 |
| Average (%) | | | | | 80.3 | | | 100 | | |
